# Supplementary material for: Integrated multiomics of pressure overload in the human heart prioritizes targets relevant to heart failure
Source: Nat Commun. 2025 Jul 26;16:6889. doi: 10.1038/s41467-025-62201-2 (PMC12297671; doi:10.1038/s41467-025-62201-2)

Integrated multiomics of pressure overload in the human heart prioritizes targets relevant to heart failure

SUPPLEMENT

Supplemental Figure 1: Principal component analysis identifies 3 components that account for ~65% of the variance explained in 12 echocardiographic measures of cardiac structure and function.

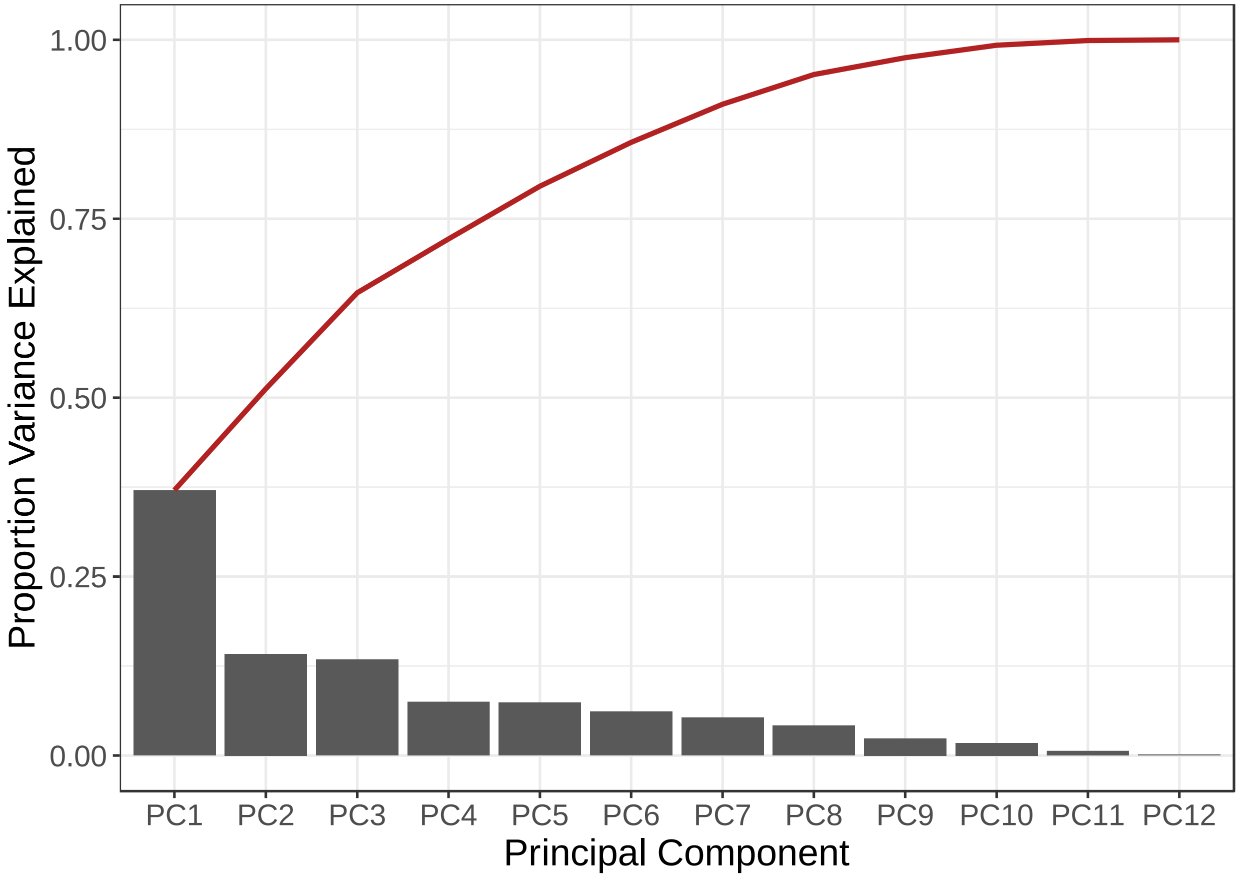

Supplemental Figure 2: Proteomic relations to cardiac remodeling. (A, B, C) Volcano plots representing results from linear models related individual proteins to each phenotype component, adjusted for age and sex. (D) Venn diagram showing the intersection of proteins with an FDR < 0.05 for each phenotype component.

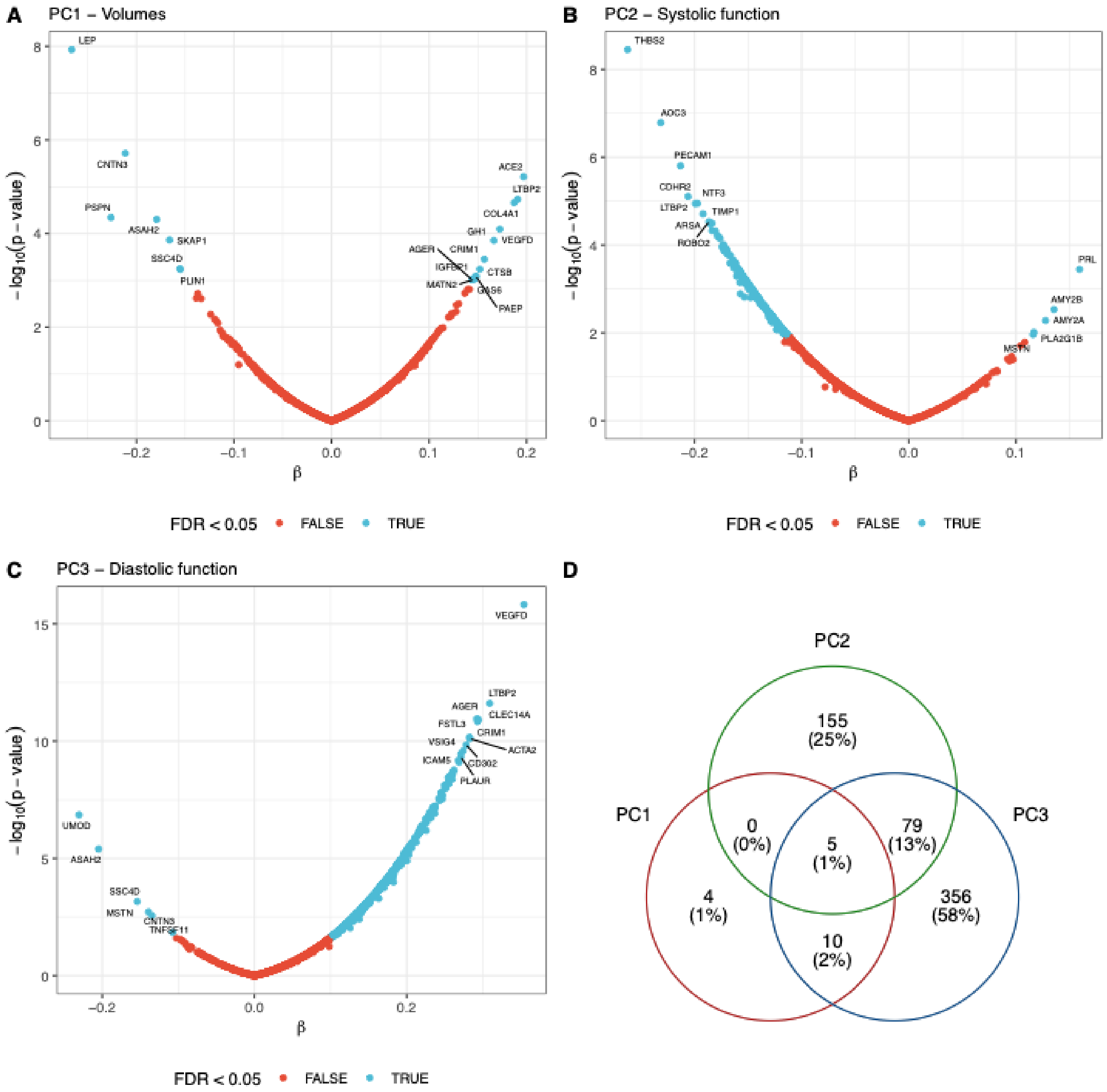

Supplemental Figure 3: Model fit of proteomic scores in derivation samples. Proteomic scores of remodeling are moderately correlated with their respective phenotype component in the AS Biomarker Cohort Derivation sample. Pearson correlation presented.

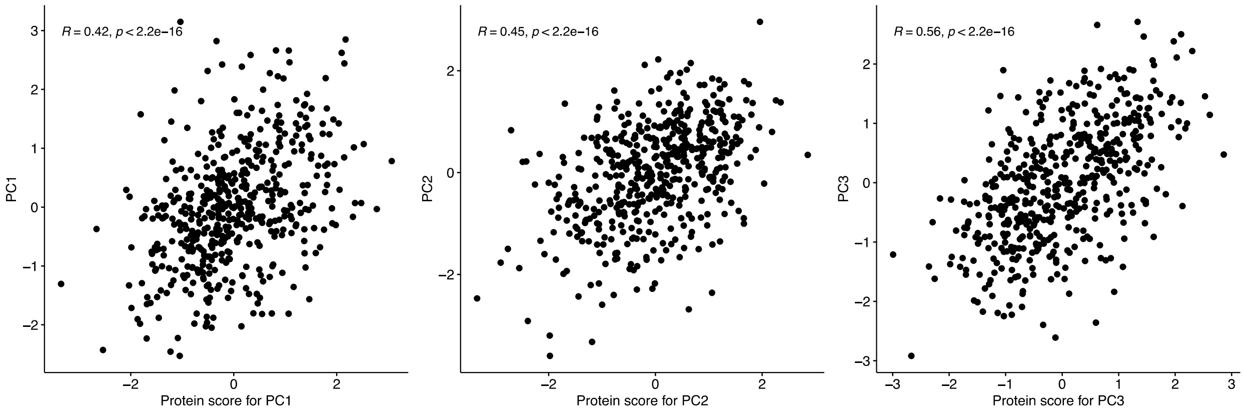

14 proteins | 16 proteins | 49 proteins

Supplemental Figure 4: Correlation of proteomic scores of remodeling with echocardiographic parameters in validation samples. In the AS Biomarker cohort validation sample (N=306), due to missingness of echocardiographic data we were unable to generate PC based scores for the remodeling phenotypes. Here, we present the Pearson correlations of the proteomic scores with the echocardiographic measures used to construct the 3 PCs of remodeling in the AS Biomarker cohort validation sample. *indicates nominal P value <0.05.

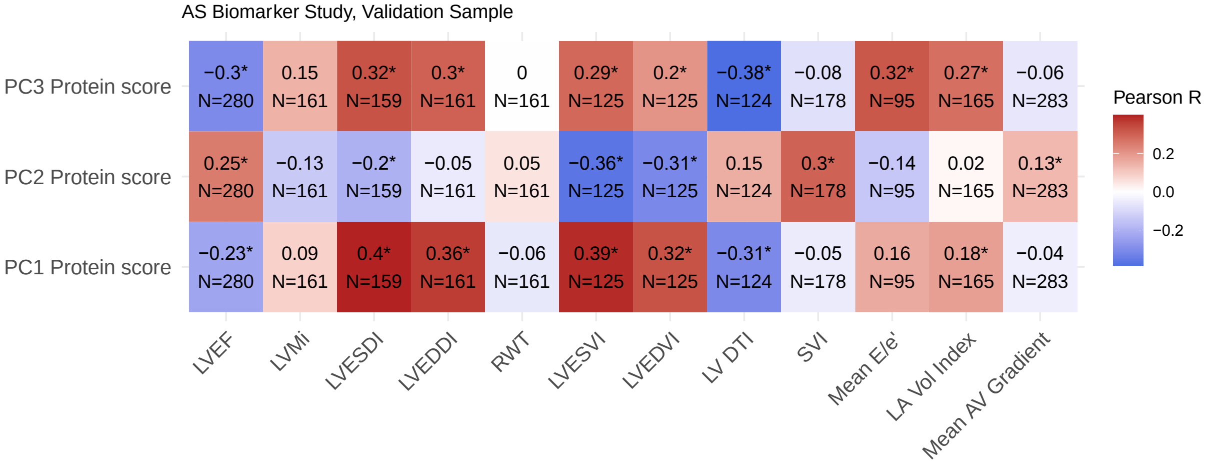

Supplemental Figure 5: Relation of proteomic scores of remodeling with age and sex. (A) Proteomic scores are weakly related to age. (B) Proteomic scores of remodeling recapitulate known sex-differences in cardiac structure and function: men tend to have larger volumes (PC1) and women tend to have higher systolic function (PC2).

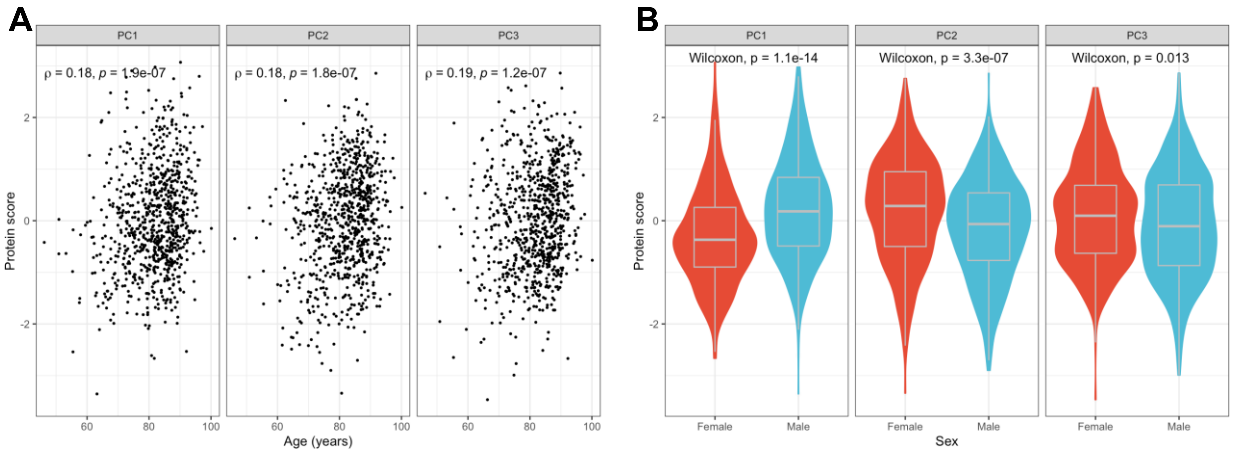

Supplemental Figure 6: Upset plot detailing the number of genes associated with remodeling (circulating proteome) which are markers of one or more major cell type. For display purposes, shared markers between cell types are limited to those on found in the most numerous cell types (e.g., not including neuronal or lymphocytes).

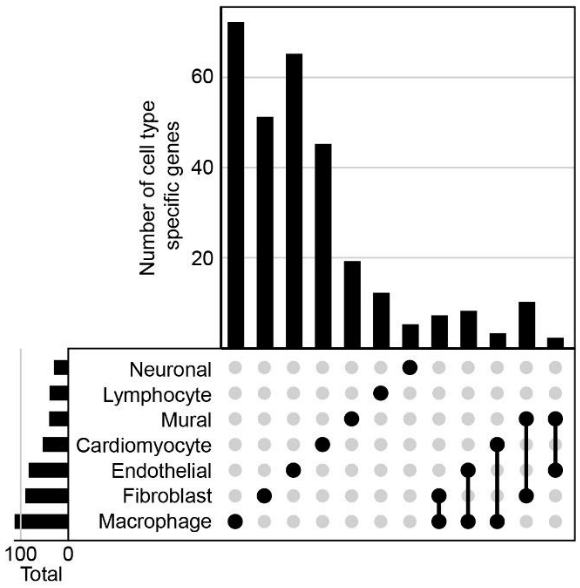

Supplemental Figure 7: Comparison between cardiomyocyte *NPPB* expression and circulating NT-proBNP levels in patients with AS.

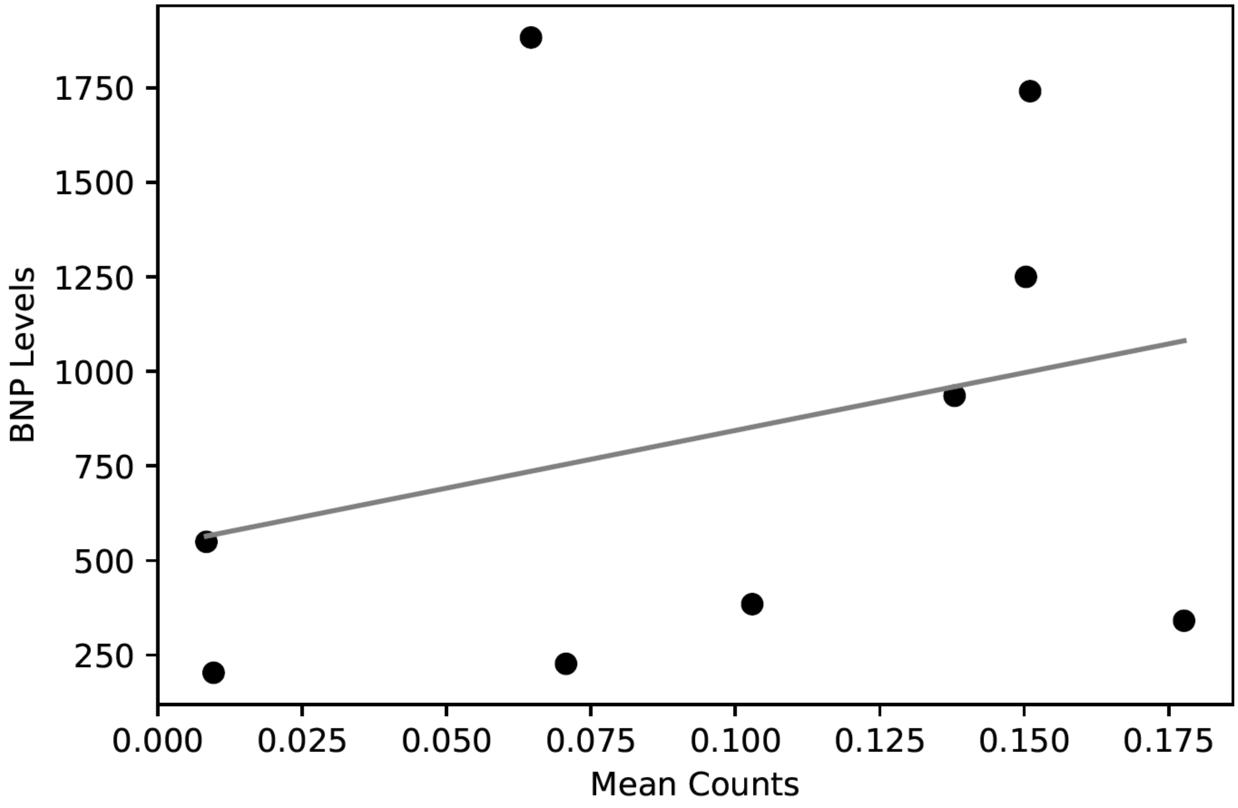

Supplemental Figure 8: UMI Decay curves for each snRNA-seq sample.

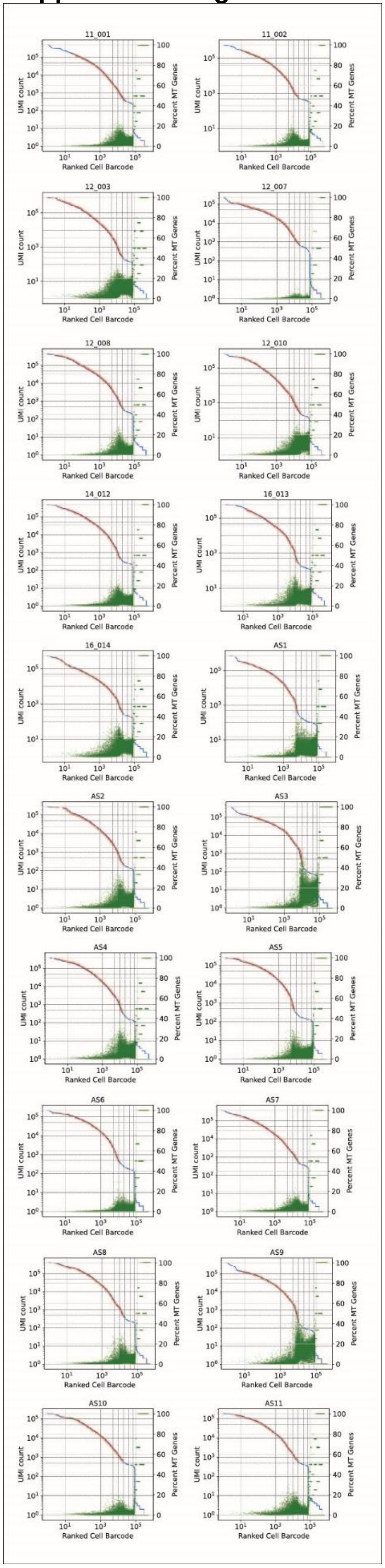

Supplemental Figure 9: Quality control plots for all snRNA-seq samples.

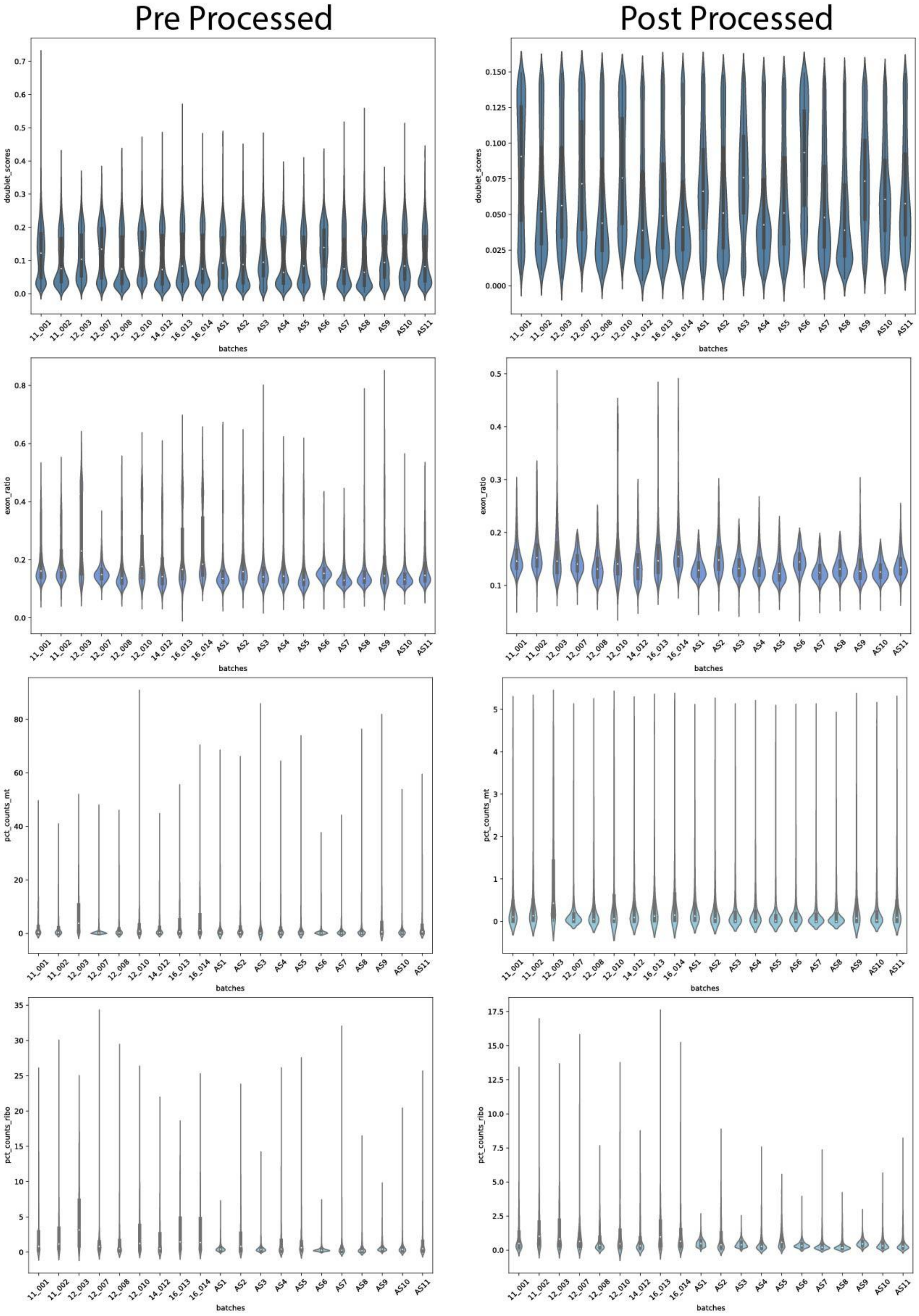

Supplement: Supplementary file 1 — Supplementary Information [file 41467_2025_62201_MOESM1_ESM.pdf]
