# Supplementary material for: Integrated multiomics of pressure overload in the human heart prioritizes targets relevant to heart failure
Source: Nat Commun. 2025 Jul 26;16:6889. doi: 10.1038/s41467-025-62201-2 (PMC12297671; doi:10.1038/s41467-025-62201-2)
Supplement: Supplementary file 2 — Description of Additional Supplementary Files [file 41467_2025_62201_MOESM2_ESM.docx]

File Name: Supplementary Data 1

Description: Regression model results relating individual proteins to principal component based measures of cardiac remodeling in the AS Biomarker Study. Model: PC ~ protein + age + sex + race.

File Name: Supplementary Data 2

Description: Regression model results relating individual proteins to cardiac magnetic resonance imaging phenotypes in the single-center CMR AS cohort. Model: outcome ~ protein + age + sex.

File Name: Supplementary Data 3

Description: LASSO model coefficients to generate the proteomic signatures of cardiac remodeling, derived in the AS Biomarker Study.

File Name: Supplementary Data 4

Description: Cox model results from the AS Biomarker Study. The outcome is all-cause death.

File Name: Supplementary Data 5

Description: Cox model results from the UK Biobank. The fully adjusted model includes age, sex, race, BMI, systolic blood pressure, diabetes, Townsend Deprivation Index, smoking, alcohol use, and LDL.

File Name: Supplementary Data 6

Description: Clinical demographics of participants whose myocardial tissue was used for single-nuclear RNA-sequencing.

File Name: Supplementary Data 7

Description: Marker genes for cell types cross referenced with significantly associated proteomics results. TRUE indicates that the gene has an AUC of greater than or equal to 0.7, with a log fold change of 0.6 or greater when compared to all other cells.

File Name: Supplementary Data 8

Description: Pseudobulk and cell-type differential expression from single-nuclear RNAseq analysis.

File Name: Supplementary Data 9

Description: Significant GO terms when using genes which were differential expressed in a cell type(s) and signficantly associated with remodeling from proteomics analysis.

File Name: Supplementary Data 10

Description: Proteome-wide association analysis results.

File Name: Supplementary Data 11

Description: Transcriptome-wide association study results.
