# Supplementary material for: Integrated multiomics of pressure overload in the human heart prioritizes targets relevant to heart failure
Source: Nat Commun. 2025 Jul 26;16:6889. doi: 10.1038/s41467-025-62201-2 (PMC12297671; doi:10.1038/s41467-025-62201-2)
Supplement: Supplementary file 4 — Reporting Summary [file 41467_2025_62201_MOESM4_ESM.pdf]

Corresponding author(s): Ravi Shah
Sammy Elmariah
Brian R. Lindman
Nathan R. Tucker

Last updated by author(s): Apr 25, 2025

Reporting Summary

Nature Portfolio wishes to improve the reproducibility of the work that we publish. This form provides structure for consistency and transparency in reporting. For further information on Nature Portfolio policies, see our Editorial Policies and the Editorial Policy Checklist.

Statistics

For all statistical analyses, confirm that the following items are present in the figure legend, table legend, main text, or Methods section.

n/a	Confirmed	
☐	☒	The exact sample size (*n*) for each experimental group/condition, given as a discrete number and unit of measurement
☐	☒	A statement on whether measurements were taken from distinct samples or whether the same sample was measured repeatedly
☐	☒	The statistical test(s) used AND whether they are one- or two-sided<br>*Only common tests should be described solely by name; describe more complex techniques in the Methods section.*
☐	☒	A description of all covariates tested
☐	☒	A description of any assumptions or corrections, such as tests of normality and adjustment for multiple comparisons
☐	☒	A full description of the statistical parameters including central tendency (e.g. means) or other basic estimates (e.g. regression coefficient) AND variation (e.g. standard deviation) or associated estimates of uncertainty (e.g. confidence intervals)
☐	☒	For null hypothesis testing, the test statistic (e.g. *F*, *t*, *r*) with confidence intervals, effect sizes, degrees of freedom and *P* value noted<br>*Give P values as exact values whenever suitable.*
☒	☐	For Bayesian analysis, information on the choice of priors and Markov chain Monte Carlo settings
☒	☐	For hierarchical and complex designs, identification of the appropriate level for tests and full reporting of outcomes
☐	☒	Estimates of effect sizes (e.g. Cohen's *d*, Pearson's *r*), indicating how they were calculated

Our web collection on statistics for biologists contains articles on many of the points above.

Software and code

Policy information about availability of computer code

Data collection	R/R Studio; Cellranger-ARC mkfastq (v2.0.0); Cutadapt (v2.8); Cellranger (v7.1.0); CellBender (v2.0); Scanpy (v1.9.3); Scrinvex (v13); scrublet; Harmonypy (v0.0.9); scCODA; edgeR
Data analysis	All code used for processing single-nuclear data and downstream analyses is deposited at https://github.com/learning-MD/Aortic_stenosis.

For manuscripts utilizing custom algorithms or software that are central to the research but not yet described in published literature, software must be made available to editors and reviewers. We strongly encourage code deposition in a community repository (e.g. GitHub). See the Nature Portfolio guidelines for submitting code & software for further information.

Data

Policy information about availability of data

All manuscripts must include a data availability statement. This statement should provide the following information, where applicable:

- Accession codes, unique identifiers, or web links for publicly available datasets
- A description of any restrictions on data availability
- For clinical datasets or third party data, please ensure that the statement adheres to our policy

Olink Proteomics data from the AS Biomarker Cohort is available at {insert figshare DOI}. Clinical data from the AS Biomarker Cohort is available upon request from the corresponding author (brian.r.lindman@vumc.org). Data from the single-center cardiac magnetic resonance (CMR) AS cohort is available upon request to Marc Dweck (Marc.Dweck@ed.ac.uk). Data from the UK Biobank is available at https://www.ukbiobank.ac.uk. All raw and processed single-nuclear RNA-sequencing data

has been deposited on NCBI GEO (GSE262690 [https://www.ncbi.nlm.nih.gov/geo/query/acc.cgi?acc=GSE262690]).

Research involving human participants, their data, or biological material

Policy information about studies with human participants or human data. See also policy information about sex, gender (identity/presentation), and sexual orientation and race, ethnicity and racism.

Reporting on sex and gender	Sex was defined as participant self-report of sex assigned at birth. We included sex as a covariate in all models. We did not include gender in our analyses as this was not felt to be a meaningful contributor to myocardial remodeling.
Reporting on race, ethnicity, or other socially relevant groupings	Race/ethnicity as used in our analyses is from participant self-report. The UK Biobank includes many categories of race/ ethnicity, we reduced the groups in our analyses to preserve degrees of freedom.
Population characteristics	The AS Biomarker Cohort included 825 participants (44% women) with severe, symptomatic aortic stenosis. The median age was 83 years and there was a high prevalence of coronary artery disease (70%) and diabetes (39%). The median left ventricular ejection fraction was 61%.<br><br>The single center CMR AS cohort included 145 participants (32% women) with mild-severe AS. The median left ventricular ejection fraction was 67%.<br><br>The UK Biobank sample consisted of 36,668 participants (54% women) representing a community based population with risk factors for heart failure: median systolic blood pressure 138mmHg, 5.7% diabetes.<br><br>The single nuclear RNA sequencing data was generated from 2 cohorts. 9 unused donor hearts (age range 23-67; 4 women) and 11 participants undergoing surgical aortic valve replacement for severe, symptomatic aortic stenosis (age range 58-95; 5 women).
Recruitment	The AS Biomarker Cohort and single center CMR cohort recruited patients from clinic and hospital based settings. The UK Biobank recruited from communities in the UK.
Ethics oversight	This study was approved by the institutional review boards at Vanderbilt University Medical Center, Massachussetts General Hospital and the University of Edinburgh. Approval for UK Biobank use is under proposal #57492.

Note that full information on the approval of the study protocol must also be provided in the manuscript.

Field-specific reporting

Please select the one below that is the best fit for your research. If you are not sure, read the appropriate sections before making your selection.

☒ Life sciences ☐ Behavioural & social sciences ☐ Ecological, evolutionary & environmental sciences

For a reference copy of the document with all sections, see nature.com/documents/nr-reporting-summary-flat.pdf

Life sciences study design

All studies must disclose on these points even when the disclosure is negative.

Sample size	As this was a discovery effort, we included all available samples from each study population available.
Data exclusions	Participants were excluded due to missing data on model predictors, covariates or outcomes.
Replication	Relationships between proteins and cardiac remodeling were performed in 2 cohorts. The clinical outcomes analyses were performed in 2 separate cohorts. Overall, our efforts to replicate the findings were successful.
Randomization	Randomization was not performed.
Blinding	Blinding was not performed.

Reporting for specific materials, systems and methods

We require information from authors about some types of materials, experimental systems and methods used in many studies. Here, indicate whether each material, system or method listed is relevant to your study. If you are not sure if a list item applies to your research, read the appropriate section before selecting a response.

Materials & experimental systems

n/a	Involved in the study
☒	☐ Antibodies
☒	☐ Eukaryotic cell lines
☒	☐ Palaeontology and archaeology
☒	☐ Animals and other organisms
☒	☐ Clinical data
☒	☐ Dual use research of concern
☒	☐ Plants

Methods

n/a	Involved in the study
☒	☐ ChIP-seq
☒	☐ Flow cytometry
☒	☐ MRI-based neuroimaging

Plants

Seed stocks	n/a
Novel plant genotypes	n/a
Authentication	n/a
